# Overconfidence, financial literacy, and panic selling: Evidence from Japan

**DOI:** 10.1371/journal.pone.0315622

**Published:** 2025-03-21

**Authors:** Aliyu Ali Bawalle, Mostafa Saidur Rahim Khan, Yoshihiko Kadoya

**Affiliations:** School of Economics, Hiroshima University, Higashihiroshima, Japan; Universiti Malaysia Sabah, MALAYSIA

## Abstract

This study investigates the phenomenon of panic selling, in which investors rapidly sell financial securities due to fear or uncertainty, often causing asset prices to plummet. Despite the significance of this behavior during market crises, there is limited understanding of the factors that drive panic selling. To address this gap, we applied the overreaction hypothesis, integrating financial literacy to explore whether investor sentiment influences trading decisions even when financial knowledge is present. We analyzed individual investor level data from a survey conducted by Rakuten Securities Company and Hiroshima University in Japan, covering the period between November and December 2023. Using probit regression models, we examined the relationship between panic selling, financial literacy, and overconfidence, while controlling for demographic, socioeconomic, and psychological factors. Our findings reveal that overconfident investors are more likely to engage in panic selling during market downturns, even when financial literacy reduces such tendencies. This relationship persists across various degrees of selling, including partial and full divestment of stocks and mutual funds. The results underscore the importance for policymakers to monitor the dissemination of negative information during crises to mitigate panic selling. At the household level, the findings highlight the need for investors to acquire financial knowledge and seek reliable information to make informed decisions, thereby reducing their susceptibility to panic selling.

## 1. Introduction

Panic selling involves the irrational and rapid disposal of stocks by investors due to fear of a declining market. This behavior often results in significant market volatility, loss of investor wealth, and disruptions in financial markets, ultimately affecting the broader economy [1,2]. Investors engage in panic selling when they anticipate further losses, preferring to sell off assets prematurely rather than holding onto them for potential recovery [3]. The economic impact of this phenomenon extends beyond individual losses—panic selling triggers large-scale market disruptions, contributing to systemic risks and destabilizing financial markets during periods of crisis [2,4]. Behavioral finance theories, such as the investor overreaction hypothesis, explain this behavior by attributing it to cognitive biases and emotional responses, which lead investors to overreact to market downturns and negative information [5–8]. As these irrational behaviors aggregate, they can exacerbate market instability, harming not only individual investors, but also institutions and the overall economy.

Recent evidence indicates that financial literacy plays a crucial role in reducing irrational investment decisions, including panic selling [9–11]. Financially literate individuals are more likely to engage in rational investment strategies and avoid emotionally driven decisions during market downturns. Financial literacy provides investors with the knowledge and skills to assess market conditions, manage risks, and maintain diversified portfolios, helping mitigate the negative effects of market volatility. Studies have shown that financially literate investors are less likely to succumb to fear during market declines and instead adopt long-term strategies that align with their financial goals [10,12–15]. Despite the advantages of financial literacy, overconfidence in one’s financial knowledge can paradoxically lead to irrational behavior, such as overtrading and panic selling. Overconfidence arises when investors overestimate their ability to interpret market signals and make decisions based on perceived rather than actual knowledge. This phenomenon, known as financial literacy overconfidence, leads investors to believe that their decisions are superior, causing them to overreact to information and engage in impulsive trading [16].

Overconfidence has been well-documented as a cognitive bias that leads investors to make suboptimal decisions in various contexts. Gervais and Odean [17] argue that overconfident investors tend to overestimate their ability to predict market movements and interpret signals, often leading to excessive trading. Such investors attribute past successes disproportionately to their own skill rather than external factors, reinforcing their overconfidence. This overestimation of one’s abilities results in overprecision and miscalibration, leading to decisions that ultimately harm portfolio performance, such as panic selling during market declines. Overconfident investors may engage in excessive trading, believing that they possess superior information, only to incur losses due to their inability to accurately forecast market trends [17–21].

Financial literacy overconfidence is a specific form of this broader cognitive bias, where individuals with higher perceived financial knowledge believe they are more capable of making sound financial decisions than they actually are. This overconfidence can distort decision-making, as investors are more likely to engage in high-risk activities, such as overtrading and reacting impulsively to negative market news [22]. Studies have shown that financial literacy overconfidence encourages risk-taking behavior, leading to higher transaction costs and lower trading profits [16,23–29]. In the context of market downturns, this overconfidence may drive investors to panic sell their stocks in response to perceived risks, exacerbating market volatility and individual losses.

While previous research has examined the role of financial literacy in promoting rational investment choices, few studies have explored how financial literacy overconfidence influences panic selling behavior. Bucher-Koenen and Ziegelmeyer [10] found that individuals with low financial literacy are more likely to panic sell during financial crises, as they are less equipped to manage the emotional and cognitive challenges posed by declining markets. In contrast, financially literate investors are generally more resilient during crises, as they rely on their knowledge to make informed decisions. However, Bucher-Koenen and Ziegelmeyer [30] also observed that financially literate investors sometimes exhibit the disposition effect, where they hold onto losing stocks for too long, which complicates the understanding of their behavior during market declines. This raises a crucial question: does overconfidence in financial knowledge override the rational tendencies of financially literate investors, leading them to panic sell during crises?

To address this gap, we investigate the association between panic selling and financial knowledge overconfidence in the context of financial literacy. Although financial literacy is often regarded as a safeguard against irrational behavior, overconfidence in one’s financial knowledge may act as a countervailing force that leads to impulsive decisions, especially during periods of market turmoil. Financial literacy overconfidence, which reflects the overestimation of one’s financial knowledge relative to actual competence, may lead to the overreaction to negative information and increased propensity to panic sell. This study builds on previous research by integrating behavioral finance theories with financial literacy frameworks to explore how these dynamics unfold in the context of significant market declines.

The contribution of this study lies in providing a comprehensive understanding of how overconfidence in financial knowledge interacts with financial literacy to influence panic selling behavior. While financial literacy is often associated with rational decision-making, our research aims to shed light on how overconfidence in financial knowledge may undermine these benefits, leading to irrational decisions during market downturns. By exploring this interaction, we contribute to the broader literature on behavioral finance, particularly the role of cognitive biases in shaping investment behavior. Furthermore, our findings have important implications for policymakers, financial educators, and market regulators. Understanding how financial literacy overconfidence influences investor behavior can inform the development of targeted interventions aimed at mitigating panic selling and promoting market stability during times of crisis.

In this study, we hypothesize that financial literacy reduces the tendency to panic sell by equipping investors with the knowledge to make informed decisions during market declines. However, financial knowledge overconfidence may cause investors to overreact to negative information, leading to impulsive selling decisions. By investigating these dynamics, we aim to contribute to the literature by bridging the gap in applying the financial literacy overreaction hypothesis to explain panic selling behavior. This research has the potential to improve our understanding of investor psychology, improve financial education programs, and ultimately contribute to a more stable and resilient financial market system.

## 2. Literature review

Several behavioral finance theories have sought to explain the phenomenon of panic selling, emphasizing its importance and its broad implications for the overall economy. For example, the Prospect Theory attributes panic selling to cognitive biases such as loss aversion, where investors irrationally prioritize avoiding losses over pursuing potential gains [31]. Similarly, the Investor Overreaction Hypothesis explains panic selling as a reaction to short-term negative information, leading to impulsive asset liquidation [5,32,33]. These theories underscore the emotional and psychological triggers that cause investors to deviate from rational decision-making, resulting in significant market volatility, loss of wealth, and systemic risk [2].

Although behavioral theories provide valuable insights into panic selling, there has been limited exploration of the role of financial literacy—an established proxy for rational decision-making—in this context. Financial literacy is often associated with better investment choices and a greater likelihood of avoiding emotional responses during market downturns [9,34]. Bucher-Koenen & Ziegelmeyer [10] found that financial literacy improves investors’ ability to assess long-term market trends, reducing the likelihood of irrational sell-offs during downturns. Yeh & Ling [26] found that individuals with higher confidence in their financial literacy are more likely to participate in stock market activities, suggesting that enhancing financial literacy and confidence can lead to more informed financial decisions and better retirement outcomes. Similarly, findings by Lusardi & Mitchell [9] and Klapper [11] show that financially literate individuals are less likely to panic sell and more likely to make informed investment decisions, particularly during financial crises. These individuals are more likely to hold onto their assets during volatile periods, trusting that the market will eventually recover. For example, Putri [14] found that financially literate investors continued to make rational decisions even during market downturns, contrasting with those with lower financial literacy, who may panic sell out of fear of further losses.

However, the protective influence of financial literacy is not absolute. When financial literacy is coupled with overconfidence, it can paradoxically lead to poor investment choices. Overconfident investors, even if who are financially literate, may overestimate their ability to interpret market signals, making them prone to overtrading during turbulent times despite their knowledge [17]. Overconfident investors may believe they have superior knowledge, leading to excessive trading or overreacting to market fluctuations. In the context of market downturns, overconfidence can distort decision-making, as investors are more likely to engage in high-risk activities such as overtrading and reacting impulsively to negative market news. Inghelbrecht & Tedde [16] found that financial knowledge overconfidence leads to increased trading activity, higher transaction costs, and lower overall returns. Cupák et al. [24] investigated the relationship between confidence, financial literacy, and investment in risky assets using data from the U.S. Survey of Consumer Finances. The study found that individuals with greater financial literacy and confidence are more likely to invest in risky assets, though excessive confidence may lead to suboptimal investment decisions. This research highlights the importance of balancing financial literacy with accurate self-assessment to improve investment outcomes. Xia, Wang, & Li [25] examined the role of financial literacy overconfidence in stock market participation and found that individuals who overestimate their financial knowledge are more likely to engage in the stock market, often without fully understanding the risks involved. This overconfidence can lead to suboptimal financial decisions, despite their willingness to invest. The nuanced interaction between financial literacy and overconfidence requires a deeper investigation to fully understand how even well-informed investors may succumb to irrational market behavior.

Although several studies have examined the effects of overconfidence on general investment behavior, there is a notable gap in the literature on the specific interaction between financial literacy overconfidence and panic selling. Most existing research focuses on the separate roles of financial literacy and overconfidence, without investigating how these factors jointly influence panic selling during periods of market distress [9,22,24,25]. This gap calls for further exploration of how overconfidence in financial knowledge undermines the protective effects of financial literacy, particularly during market downturns, when investor behavior is most likely to diverge from rational models.

## 2. Data and methods

### 2.1. Data

We employed the 2023 wave dataset from a survey conducted by Rakuten Securities Company and Hiroshima University in Japan (see Supplementary Information, S1 Data, for further details). The survey was administered between November and December 2023, targeting clients of Rakuten Securities Company aged 18 years and above who had purchased or invested at least once through Rakuten Securities and had logged in to their website at least once since 2020. Survey questions included the “big three financial literacy questions,” financial knowledge self-assessment, and selling behavior regarding stocks and mutual funds during the March 2020 stock market nosedive. Additionally, the questionnaire collected investors’ socioeconomic and demographic information. This approach enabled us to comprehensively examine panic-selling behavior among the Japanese population during the COVID-19 pandemic.

### 2.2 Variables

#### 2.2.1. Dependent variable.

Panic selling is typically defined in the literature as the rapid and irrational liquidation of assets, driven by fear rather than sound judgment [12,35]. De Bondt and Thaler [5] describe panic selling as part of an investor’s overreaction to short-term negative information, which results in impulsive stock sell-offs. Baker et al. [7] and Elkind et al. [35] operationalize it as an event in which investors sell a significant portion (over 80%) of their equity holdings during market downturns, driven by fear of loss. Similarly, Kindleberger and Aliber [2] characterize panic selling as a mass liquidation of assets during financial crises, which exacerbates market volatility. Lusardi and Mitchell [9] further associate panic selling with behavioral biases, such as loss aversion, where investors prioritize avoiding losses, even at the expense of long-term gains.

In line with this theoretical background and the objectives of this study, we define panic selling as the complete or partial liquidation of stocks. The complete liquidation of the stock was used as the primary indicator of panic selling. In the survey, respondents were asked to choose from the following options regarding their management of stocks and mutual funds (including ETFs) during the March 2020 market downturn caused by the COVID-19 pandemic:

1. Sold part of the stock or mutual fund
2. Sold all stocks and mutual funds
3. Increased purchase of stocks and mutual funds
4. Took the opportunity to buy stocks and mutual funds for the first time
5. No changes to my investment amount in stocks and mutual funds (continued investing as I before)
6. Held stocks and mutual funds but did not buy or sell during this period
7. Did not invest in or hold stocks or mutual funds at the time

Based on these responses, a binary dependent variable, “tot_panic_selll,” was created, where a value of 1 was assigned if the respondent sold all their stocks and investment trusts, and 0 if they did not. To ensure the robustness of the findings, a more flexible definition of panic selling, partial stock sales, was adopted. A second binary variable, “part_panic_sell,” was introduced, with a value of 1 assigned if the respondents sold part of their stocks and investment trusts, and 0 otherwise.

#### 2.2.2. Independent variables.

We focus on financial literacy and financial knowledge overconfidence. Following previous studies, we define financial literacy as a set of knowledge, skills, attitudes, and behaviors that enable individuals access, process, and use economic information, leading to the effective utilization of economic resources and financial well-being [36,37]. We consider financial knowledge overconfidence as a mismatch between objective and subjective assessments of one’s financial knowledge, aligning with Alba and Hutchinson’s [38] definition of overconfidence as a disagreement (miscalibration) between objective and subjective knowledge assessments.

Additionally, the remaining independent variables comprise respondents’ gender, age, education, marital status, child(ren), household income, household assets, risk aversion, and myopic view of life. Detailed definitions of the variables are provided in Table 1.

**Table 1:** Definitions and measurement of variables.

Dependent Variables	
Tot_Panic_Sell (Total sale of stocks or mutual funds)	1 if the respondent sold all stocks or mutual funds during the COVID-19 pandemic (March 2020), and 0 otherwise
Part_Panic_Sell (Partial sale of stocks or mutual funds)	1 if the respondent partially sold stocks or mutual funds during the COVID-19 pandemic (March 2020), and 0 otherwise
Cons Investor (Maintaining Investment Portfolio)	1 if the respondent did not change stocks or mutual funds portfolio during the COVID-19 pandemic (March 2020), and 0 otherwise
Independent Variables	
Fin_literacy (Financial Literacy)	Average Score of Big Three Financial Literacy Questions
Over_Conf (Financial Knowledge Overconfidence)	1 if the respondent scored below average in financial literacy questions but are confident about his/her financial knowledge, and 0 otherwise
Under_Conf (Financial Knowledge Underconfidence)	1 if the respondent scored above average in financial literacy questions but are not confident about his/her financial knowledge, and 0 otherwise
Gender	1 if male and 0 Otherwise
Age	Age of respondents
AgeS	Squared Age of Respondents
Marital status	1 if the respondent is married, and 0 otherwise
Children	1 if the respondent has a child, and 0 otherwise
Uni_Educ University Degree	1 if the respondent has a university degree, and 0 otherwise
Employment	1 if the respondent is employed, and 0 otherwise
lH_Income	Natural log of household estimates annual gross income including taxes in 2023
lH_Asset	Natural log of household net financial asset
Risk_Aversion	Measure of the respondent’s risk taking, ranging from 0 to 1
Myopic_View	Ordinal variable range 1–5
Urban Dummy	1 if the respondent lives in an urban area, and 0 otherwise

### 2.3. Measuring financial literacy and financial knowledge overconfidence

Using “the big three financial literacy questions” in the survey, we followed the methodology of Lusardi & Mitchell [39] to measure respondents’ financial literacy. The survey questions are as follows.

1. Suppose you have ¥100 in a savings account with a 2% annual interest rate, and you never withdraw money or interest payments. After five years, how much would you have in this account in total? Optional answers: More than ¥102, Exactly ¥102, Less than ¥102, and do not know.
2. If the interest rate on your savings account is 1% annually and inflation is 2% annually, how much can you buy with the money in this account after one year? Optional answers: More than today, exactly the same, less than today, and do not know.
3. Please indicate whether the following statement is true or false. “Buying a company stock usually provides a safer return than a stock mutual fund.” Optional answers: True, false, and do not know.

The first and second questions measured respondents’ grasp of basic economic concepts (interest and inflation) and their ability to perform basic numerical computations. The third question assessed respondents’ knowledge of stocks, mutual funds, and investment risk diversification. To measure respondents’ financial literacy, we calculated a simple average of correct answers from these three questions, a method used in several other studies [37,40].

Financial knowledge overconfidence measures investors’ overly optimistic perceptions of their financial knowledge. Overconfident investors often believe that they possess more knowledge and skills than others. Existing literature on financial literacy overconfidence considers the difference between objective and subjective financial knowledge as a benchmark for overconfidence [23,25,26]. Similarly, in our survey, we used a statement querying respondents’ confidence in their financial knowledge (subjective financial knowledge): “*I am confident about my financial knowledge.”* Response options were (1) totally agree, (2) somewhat in favor, (3) I cannot say either way, (4) rather the opposite, and (5) completely disagree*.* We measured financial knowledge overconfidence using respondents’ answers to this question and their performance on the objective financial literacy question. Specifically, respondents who selected either (1) “totally agree” or (2) “somewhat in favor” and scored below average in objective financial literacy were considered overconfident. It is worth noting that this method offers a better measure of respondents’ financial knowledge overconfidence, as it is constructed from the average objective financial literacy score and respondents reported financial knowledge confidence.

### 2.4. Descriptive Statistics

Descriptive statistics for the study variables are presented in Table 2. The results show that the behavioral variables such as Tot_Panic_Sell and Part_Panic_Sell have relatively low mean values (0.044 and 0.012, respectively), indicating that only a small fraction of the sample engaged in panic selling. However, both variables show strong right skewness (4.431 and 8.81) and high kurtosis (20.634 and 78.623), suggesting the presence of outliers where a few respondents exhibited extreme behaviors. Similarly, Overconfidence (mean of 0.07) is rare but shows strong positive skewness (3.359) and leptokurtosis (12.283), indicating that most respondents were not overconfident, but a small group showed extreme overconfidence. In contrast, Underconfidence is more common, with a mean of 0.369 and moderate skewness (0.543), implying a broader distribution of responses, though it is still slightly skewed toward lower values.

**Table 2:** Descriptive statistics.

Variables	Mean	Std. Dev.	Min	Median	Max	Skew.	Kurt.
Tot Panic Sell	0.012	0.111	0	0	1	8.81	78.623
Part Panic Sell	0.044	0.206	0	0	1	4.431	20.634
Cons Investor	0.229	0.42	0	0	1	1.293	2.671
Fin literacy	0.782	0.305	0	1	1	-1.253	3.511
Over Conf	0.07	0.256	0	0	1	3.359	12.283
Under Conf	0.369	0.483	0	0	1	0.543	1.295
Gender	0.643	0.479	0	0	1	-0.596	1.355
Age	45.255	12.249	18	45	94	0.285	2.46
AgeS	2198.095	1164.794	324	2025	8836	0.836	3.362
Married	0.669	0.471	0	1	1	-0.716	1.513
Children	0.451	0.498	0	0	1	0.199	1.039
Uni Educ	0.638	0.481	0	1	1	-0.575	1.33
Employment	0.936	0.245	0	1	1	-3.564	13.7
H Income	7,450,000	4,160,000	1,000,000	7,000,000	20,000,000	1.016	3.965
H Asset	19,200,000	23,700,000	2,500,000	8,750,000	100,000,000	2.013	6.325
Risk Aversion	0.535	0.231	0	0.5	1	0.007	2.795
Myopic View	2.424	0.966	1	2	5	0.392	2.56
Urban	0.158	0.365	0	0	1	1.872	4.505
Observations: 191,762							

Demographic variables like gender show that 64.3% of the respondents are male, with a slight negative skew (-0.596) and near-normal kurtosis (1.355). The mean age of respondents is 45.25 years, with a slight positive skew (0.285) and mildly leptokurtic distribution (2.46). Similarly, variables such as Married (mean of 0.669) and Children (mean of 0.451) show distributions that are moderately skewed and relatively close to normal in kurtosis, reflecting typical population distributions for these traits. Educational attainment, captured through University Education (mean of 0.638), shows a small negative skew (-0.575), suggesting a slightly higher proportion of university graduates in the sample.

Financial variables such as household income and household assets are both positively skewed (1.016 and 2.013, respectively), with high kurtosis values (3.965 and 6.325), indicating that while most respondents have moderate levels of income and assets, a small number of respondents report very high values, thus creating a heavy tail in the distribution. The mean household income is ¥7,450,000, and household assets average ¥19,200,000, with large variability as indicated by their standard deviations.

Finally, psychological and behavioral variables like Risk Aversion (mean of 0.535) and Myopic View (mean of 2.424) demonstrate relatively symmetric distributions, with minimal skewness (0.007 and 0.392, respectively) and kurtosis values close to normal (2.795 and 2.56). The urbanization variable (Urban) shows that 15.8% of the respondents live in urban areas, with a strong positive skew (1.872), indicating that most respondents are from rural or non-urban regions. Overall, the dataset provides a diverse set of variables with varying degrees of skewness and kurtosis, highlighting the presence of both typical and extreme behaviors and characteristics within the sample of 191,762 observations.

### 2.5. Methods

We used the probit model regression to investigate the relationship among panic-selling behavior during the COVID-19 pandemic, financial knowledge overconfidence, and financial literacy. We created four models to estimate how financial literacy and financial knowledge overconfidence correlate with the partial or total sale of stocks or mutual funds while maintaining portfolio positions. In Models 1 and 2, total sale of stocks and mutual funds serve as the dependent variable. Model 1 considers financial literacy as the main independent variable, while Model 2 incorporates both financial literacy and overconfidence. Similarly, in Models 3 and 4, partial sale of stocks and mutual funds serve as the dependent variable, with financial literacy as the main independent variable in Model 3 and both financial literacy and overconfidence in Model 4. Additionally, gender, age, education, marital status, children, household income, household assets, risk aversion, and a myopic view of life are included as explanatory variables. The model specifications are presented below.

To avoid multicollinearity among the explanatory variables, we conducted a multicollinearity test across all models. For example, educated respondents may exhibit higher financial literacy and income, household income could be linked to household financial assets, age may relate closely to age squared and having children, and being married could be linked to having children. Our results reveal the absence of multicollinearity (variance inflation factors are below 10), as presented in Appendix A.


$$\begin{array}{l} \text{Tot}\_\text{Panic}\_{\text{Sell}}_{i}=\beta_{0}+\beta_{1}Fin\_literacy_{1}+\beta_{2}Gender_{2}+\beta_{3}Age_{3}+\beta_{4}AgeS_{4}+\\ \beta_{5}Married_{5}+\beta_{6}Childreen_{6}+\beta_{7}Uni_{Educ}{}_{7}+\beta_{8}lH_{Income}{}_{8}+\beta_{9}lH_{Asset}{}_{9}+\\ \beta_{10}Risk_{Aversion}{}_{10}+\beta_{11}Myopic_{View}{}_{11}+\beta_{12}Urban_{Dummey}{}_{12}+\varepsilon_{i} \end{array}$$ (1)

$$\begin{array}{l} \text{Tot}\_\text{Panic}\_{\text{Sell}}_{i}=\beta_{0}+\beta_{1}Fin\_literacy_{1}+\beta_{2}\text{Over}\_{\text{Conf}}_{2}+\beta_{3}Gender_{3}+\beta_{4}Age_{4}+\\ \beta_{5}AgeS_{5}+\beta_{6}Married_{6}+\beta_{7}Childreen_{7}+\beta_{8}Uni\_Educ_{8}+\beta_{9}lH\_Income_{9}+\\ \beta_{5}AgeS_{5}+\beta_{6}Married_{6}+\beta_{7}Childreen_{7}+\beta_{8}Uni\_Educ_{8}+\beta_{9}lH\_Income_{9}+ \end{array}$$ (2)

$$\begin{array}{l} \text{Part}\_\text{Panic}\_{\text{Sell}}_{i}=\beta_{0}+\beta_{1}Fin_{literacy}{}_{1}+\beta_{2}Gender_{2}+\beta_{3}Age_{3}+\beta_{4}AgeS_{4}+\beta_{5}Married_{5}+\\ \beta_{6}Childreen_{6}+\beta_{7}Uni_{Educ}{}_{7}+\beta_{8}lH_{Income}{}_{8}+\beta_{9}lH_{Asset}{}_{9}+\beta_{10}Risk_{Aversion}{}_{10}+\\ \beta_{11}Myopic_{View}{}_{11}+\beta_{12}Urban_{Dummey}{}_{12}+\varepsilon_{i} \end{array}$$ (3)

$$\begin{array}{l} \text{Part}\_\text{Panic}\_{\text{Sell}}_{i}=\beta_{0}+\beta_{1}Fin\_literacy_{1}+\beta_{2}\text{Over}\_{\text{Conf}}_{2}+\beta_{3}Gender_{3}+\beta_{4}Age_{4}+\\ \beta_{5}AgeS_{5}+\beta_{6}Married_{6}+\beta_{7}Childreen_{7}+\beta_{8}Uni\_Educ_{8}+\beta_{9}lH\_Income_{9}+\\ \beta_{10}lH\_Asset_{10}+\beta_{11}Risk\_Aversion_{11}+\beta_{12}Myopic\_View_{12}+\beta_{13}Urban\_Dummey_{13}+\varepsilon_{i} \end{array}$$ (4)

### 2.6. Ethics statement

Review and approval by an ethics committee was not needed for this study because this study does not involve animal experiments, human and behavior studies. However, the authors obtained written informed consent from the participants to partake in the survey.

## 3. Empirical findings

### 3.1. Regression results

Table 3 reports the results of the probit regression models estimating the association between panic selling and financial literacy and overconfidence. Models 1 and 2 report the estimation results for total panic selling, while Models 3 and 4 report the estimation results for partial panic selling. The findings suggest a significantly negative association between financial literacy and both partial and full panic selling. This indicates that financially savvy individuals are less likely to panic sell their stocks and mutual funds during market crises. Furthermore, the results reveal that, even after controlling for financial literacy, financial knowledge overconfidence is positively and significantly associated with panic selling. This suggests that overconfident individuals tend to panic sell their investments, regardless of their financial literacy. Overall, the results support our hypothesis that financial literacy reduces the likelihood of panic selling. However, investor overconfidence triggers panic selling during market crises.

**Table 3:** Probit regression results.

	Model (1)	Model (2)	Model (3)	Model (4)
	Tot_Panic_Sell	Tot_Panic_Sell	Part_Panic_Sell	Part_Panic_Sell
Fin_literacy	-0.44***	-0.423***	-0.264***	-0.245***
	(0.026)	(0.026)	(0.017)	(0.018)
Over_Conf		0.134***		0.13***
		(0.027)		(0.018)
Gender	0.454***	0.448***	0.334***	0.328***
	(0.021)	(0.021)	(0.012)	(0.012)
Age	-0.008*	-0.007	0.002	0.003
	(0.004)	(0.004)	(0.003)	(0.003)
AgeS	0.0001**	0.0001**	0.0001**	0.0001*
	(0.000)	(0.000)	(0.000)	(0.000)
Married	0.003	0.003	0.017	0.019
	(0.021)	(0.021)	(0.013)	(0.013)
Children	-0.016	-0.016	-0.008	-0.008
	(0.019)	(0.019)	(0.012)	(0.012)
Uni_Educ	-0.105***	-0.107***	0.014	0.012
	(0.018)	0(.018)	(0.011)	(0.011)
Employment	-0.041	-0.038	-0.088***	-0.085***
	(0.036)	(0.036)	(0.022)	(0.022)
lH_Income	-0.066***	-0.067***	-0.051***	-0.054***
	(0.024)	(0.024)	(0.015)	(0.015)
lH_Asset	-0.047***	-0.053***	0.016*	0.011
	(0.013)	(0.013)	(0.008)	(0.008)
Risk_Aversion	0.198***	0.196***	0.134***	0.134***
	(0.036)	(0.036)	(0.023)	(0.023)
Myopic_View	0.022**	0.022***	0.016***	0.017***
	(0.009)	(0.009)	(0.005)	(0.005)
Urban_Dummy	0.049**	0.048**	0.024*	0.023*
	(0.022)	(0.022)	(0.014)	(0.014)
_cons	-2.033***	-2.069***	-1.979***	-2.012***
	(0.103)	(0.103)	(0.068)	(0.068)
Observations	191772	191772	191772	191772
Pseudo R^2^	0.038	0.039	0.026	0.027

*Robust standard errors are in parentheses*

***p < .01,

**p < .05,

*  p < .1

Regarding the control variables, the regression results show that being male, having a myopic view of the future, being risk-averse, and living in urban areas are positively associated with panic selling. Conversely, household income is negatively associated with panic selling. Moreover, age, having a university education, and being employed exhibit an inconsistent negative association with panic selling. Finally, the association between household assets and outcome variables varies. For instance, the results show a positive association with the partial sale of stocks and mutual funds but a negative relationship with the total sale of stocks and mutual funds.

We also measured the marginal effects of probit regression models (Appendix B). The results show that a standard deviation increase in financial literacy is associated with a 2.4% decrease in the probability of partial panic selling and a 1.4% decrease in total panic selling, the results are statistically significant. Furthermore, a standard deviation increase in financial literacy overconfidence is associated with a 1.2% increase in the likelihood of partial panic selling and a 0.4% increase in the probability of total panic selling, the results are statistically different from zero. In particular, men are 3.1% more likely to engage in partial panic sales and 1.4% total panic sales than women. Risk aversion appears to increase the probability of panic selling by 1.2% percent and 0.6% of total panic sales. Additionally, employment and household income reduce the likelihood of panic selling by 0.8% percent and 0.5%, respectively. University education and household financial assets reduce the probability of total panic selling by 0.3% and 0.1%, respectively.

### 3.2. Robustness check

We tested the robustness of our results using alternative dependent and independent variables. First, we examined the stock-selling behavior of financially underconfident investors during market crises. Financially underconfident investors are those whose objective financial literacy scores are higher than their self-assessment of financial knowledge. According to the overconfidence hypothesis, financially underconfident investors should behave differently from financially overconfident investors, avoiding panic selling during a market crisis. Models 5 and 6 in Table 4 report the regression results for underconfident investors. Supporting this hypothesis, our results show a strong positive association between under-confidence and both partial and full panic selling, even after controlling for financial literacy. Second, we replaced the dependent variable “panic selling” with “consistent investment,” wherein investors avoid panic selling and continue with their usual investment strategies. Models 7 and 8 in Table 4 report the regression results for consistent investments. Our findings show that financial literacy overconfidence is not associated with “consistent investment,” suggesting that overconfident investors prefer panic selling. However, financial literacy shows a significantly positive relationship with consistent investments, while financial literacy under-confidence exhibits a significantly negative association. This indicates that financially literate and underconfident investors continue to maintain their usual investment strategies.

**Table 4:** Robustness test results.

	Model (5)	Model (6)	Model (7)	Model (8)
	Tot_Panic_Sell	Part_Panic_Sell	Cons_Investor	Cons_Investor
Fin_literacy	-0.433***	-0.246***	0.277***	0.28***
	(0.026)	(0.018)	(0.012)	(0.012)
Over_Conf			0.007	
			(0.013)	
Under_Conf	-0.049***	-0.128***		-0.023***
	(.017)	(.011)		(0.007)
Gender	0.451***	0.325***	-0.101***	-0.103***
	(0.021)	(0.012)	(0.007)	(0.007)
Age	-0.007*	0.003	0.017***	0.017***
	(0.004)	(0.003)	(0.002)	(0.002)
AgeS	0.0001**	0.0001*	0.0001***	0.0001***
	(0.000)	(0.000)	(0.000)	(0.000)
Married	0.003	0.018	0.046***	0.046***
	(0.021)	(0.013)	(0.008)	(0.008)
Children	-0.017	-0.009	0.01	0.01
	(0.019)	(0.012)	(0.007)	(0.007)
Uni_Educ	-0.105***	0.013	0.06***	0.06***
	(0.018)	(0.011)	(0.007)	(0.007)
Employment	-0.039	-0.084***	-0.044***	-0.043***
	(0.036)	(0.022)	(0.015)	(0.015)
lH_Income	-0.066***	-0.051***	0.005	0.005
	(0.024)	(0.015)	(0.009)	(0.009)
lH_Asset	-0.051***	0.006	0.034***	0.032***
	(0.013)	(0.008)	(0.005)	(0.005)
Risk_Aversion	0.197***	0.133***	0.018	0.018
	(0.036)	(0.023)	(0.014)	(0.014)
Myopic_View	0.022**	0.016***	-0.001	-0.001
	(0.009)	(0.005)	(0.003)	(0.003)
Urban_Dummy	0.049**	0.023*	-0.014	-0.014
	(0.022)	(0.014)	(0.009)	(0.009)
_cons	-2.02***	-1.946***	-1.509***	-1.502***
	(0.103)	(0.068)	(0.043)	(0.043)
Observations	191772	191772	191772	191772
Pseudo R^2^	0.039	0.028	0.009	0.009

*Robust standard errors are in parentheses*

***p < .01,

**p < .05,

*  p < .1

## 4. Discussion

To address the lack of explanation for why investors panic sell during market crises, we employed the overreaction hypothesis, which has been previously used to explain the investment patterns of irrational investors. Additionally, we incorporated financial literacy into the overreaction hypothesis to ascertain whether rational investors also succumb to panic selling. In support of our hypothesis, our findings demonstrate that overconfident investors are more likely to panic sell during crises, even after controlling for investors’ financial literacy levels. We validated these findings through robustness checks using alternative dependent and independent variables, which consistently supported the overreaction hypothesis. Consistent with this hypothesis, we posit that investors often overreact to negative information during market crises because of overprecision and miscalibration. Overconfident investors tend to rely on their existing knowledge than seeking new information, leading to suboptimal efforts in information gathering [38]. Consequently, they engage in panic selling to avoid further losses and regret. Our results align with those of previous studies that have shown higher trading activity and lower portfolio performance among overconfident investors [18–21,38]. Furthermore, our findings support the notion of irrational trading practices among financially literate, overconfident investors [10,16,30]. Additional factors contributing to this behavior include the propensity for aggressive trading and self-managed investment portfolios. This aligns with Chu et al. [23] and Kramer [41], who suggested that overconfident investors tend to manage their investment portfolios independently than relying on financial advisors.

Panic selling is often cited as evidence of irrational behavior among overconfident investors. However, our study reveals a noteworthy correlation: financially literate individuals demonstrate a decreased tendency toward panic selling. Our results align with those of Bucher-Koenen and Ziegelmeyer [10], who argue that individuals with robust financial knowledge are better equipped to understand and effectively process financial information. Consequently, they are more likely to evaluate market signals prudently and respond rationally. Notably, Zoé [28] highlights that market interactions involve intricate tasks, necessitating a comprehensive understanding of financial market dynamics. These cognitive demands were exacerbated during the pandemic, highlighting the critical link between financial literacy and cognitive capacity.

Regarding the control variables, our findings suggest that men exhibit a greater inclination toward panic selling during the pandemic and are more prone to deviate from their pre-pandemic investment patterns. This observation aligns with Elkind et al. [35], who attribute irrational male behavior to the gender overconfidence gap. Given that men typically exhibit higher confidence levels, overconfidence may drive more active trading. Additionally, aligning with the findings of Kato [42], household income is negatively associated with panic selling. This may be because higher-income households, which generally have higher cognitive abilities, are better equipped to navigate market volatility. Age demonstrates an inverted U-shaped effect on the total sale of stocks or mutual funds during the pandemic, and a U-shaped effect on investment consistency. This suggests that age tends to mitigate panic selling during financial uncertainty. However, the likelihood of panic selling increases with age, aligning with Zhou [43] and Kato [42]. Furthermore, our results indicate a negative association between education and panic selling, in line with Zhou [43]. As education is positively related to financial literacy, individuals with higher education levels are better positioned to understand market dynamics and the consequences of panic trading. Moreover, risk aversion and a myopic view of the future exhibit positive correlations with panic selling, indicating that impulsive, short-term-oriented, and risk-averse investors are more likely to engage in panic selling. Finally, residing in urban areas and being employed are positively associated with panic selling. We attribute this behavior to the information overload effect, which becomes pronounced during market crises [44].

## 5. Conclusions

Panic selling during market crises is a long-term phenomenon; it profoundly influences household portfolio management and disrupts the smooth functioning of financial markets. It alters existing portfolios and deters impacts investors from returning to the market. Thus far, explanations for panic selling have primarily focused on investors’ tendencies toward loss and regret aversion. We build on theories of overconfidence and its effects on trading behavior, suggesting that during crises, investors’ overreaction to negative information triggers panic selling. Additionally, we propose that financial literacy independently influences panic selling. However, during market crises, overconfidence may overshadow its effects.

Using data obtained from a collaborative survey by Rakuten Securities Company and Hiroshima University, Japan, we employed probit regression models to examine the relationship between panic selling and both financial literacy and overconfidence, while controlling for demographic, socioeconomic, and psychological factors. Our findings support our hypotheses, revealing a positive association between investor overconfidence and panic selling. Importantly, this relationship persists even after accounting for the negative association between panic selling and financial literacy. These results hold for various degrees of selling, including partial or full divestment of stocks and mutual funds. Additionally, we verified the robustness of our findings using alternative definitions and measures of panic selling and overreaction, confirming their reliability.

Our findings offer valuable insights for policymakers and households to address panic selling. First, policymakers should expand financial literacy programs, particularly during stable times, to prepare investors for future crises. These programs should cover not only basic financial concepts but also psychological biases such as overconfidence and loss aversion, which influence behavior in volatile markets. Second, during downturns, governments should actively manage the spread of negative information, providing accurate guidance to counter fear-driven narratives and encouraging professional financial advice. Third, behavioral interventions can help investors make more deliberate decisions. For example, platforms could integrate automatic alerts or default options that discourage immediate asset liquidation, as well as “cooling-off periods” prior to large sales. Finally, policymakers should promote accessible financial advisory services, especially during crises, by supporting subsidized or government-endorsed options for those lacking private advisors. At the household level, investors should improve their financial knowledge, understand market mechanisms, and seek reliable information or expert advice before making major portfolio decisions. These efforts can help mitigate panic selling and promote financial stability during crises.

While this study offers important insights, it has certain limitations. First, this study uses the identical parameters and error structures across models with different dependent variables: Total Panic Sell and Partial Panic Sell. While this unified approach allows us to identify common predictors of panic selling, it may also obscure unique influences or patterns specific to each type of selling behavior. Future studies could address this by using distinct specifications for each model to enhance predictive accuracy and capture differences between total and partial selling behaviors. Second, the data were collected exclusively from internet-based customers, which may limit the broader applicability of the findings. Third, our analysis relied on data from a single wave of panic selling, which restricts the availability of longitudinal evidence on this behavior. To address these limitations, future research should adopt more diverse data collection methods and conduct longitudinal studies to investigate how panic selling behavior evolves among hyperbolic discounters as the pandemic gradually subsides.

## Appendix A

**Table A1:** Variance Inflation Factor (VIF).

	Model 1 Part_Panic_Sell	Model 2 Part_Panic_Sell	Model 3 Tot_Panic_Sell	Model 4 Tot_Panic_Sell	Model 5 Part_Panic_Sell	Model 6 Tot_Panic_Sell	Model 7 Cons_Investor	Model 8 Cons_Investor
lH Income	1.578	1.579	1.578	1.579	1.578	1.578	1.579	1.578
Married	1.473	1.473	1.473	1.473	1.473	1.473	1.473	1.473
Age	1.472	1.472	1.472	1.472	1.472	1.472	1.472	1.472
lH Asset	1.416	1.427	1.416	1.427	1.434	1.434	1.427	1.434
Childreen	1.336	1.336	1.336	1.336	1.336	1.336	1.336	1.336
Employment	1.278	1.28	1.278	1.28	1.279	1.279	1.28	1.279
Uni Educ	1.143	1.144	1.143	1.144	1.143	1.143	1.144	1.143
Fin literacy	1.141	1.167	1.141	1.167	1.155	1.155	1.167	1.155
Gender	1.108	1.114	1.108	1.114	1.116	1.116	1.114	1.116
Under Conf	–	–	–	–	1.034	1.034		1.034
Over Conf	–	1.034	–	1.034	–	–	1.034	–
Risk Aversion	1.034	1.034	1.034	1.034	1.034	1.034	1.034	1.034
Myopic view	1.032	1.032	1.032	1.032	1.032	1.032	1.032	1.032
Urban Dummey	1.016	1.016	1.016	1.016	1.016	1.016	1.016	1.016
Mean VIF	1.252	1.239	1.252	1.239	1.239	1.239	1.239	1.239

## Appendix B

**Table A2:** Average Marginal Effects

	Part_Panic_Sell	Part_Panic_Sell	Tot_Panic_Sell	Tot_Panic_Sell	Part_Panic_Sell	Tot_Panic_Sell	Cons_Investor	Cons_Investor
Fin_literacy	-0.024 (0.002)	-0.022 (0.002)	-0.014 (0.001)	-0.013 (0.001)	-0.022 (0.002)	-0.014 (0.001)	0.083 (0.003)	0.084 (0.003)
Over_Conf		0.012 (0.002)		0.004 (0.001)			0.002 (0.004)	
Under_Conf					-0.012 (0.001)	-0.002 (0.001)		-0.007 (0.002)
Gender	0.031 (0.001)	0.030 (0.001)	0.014 (0.001)	0.014 (0.001)	0.030 (0.001)	0.014 (0.001)	-0.030 (0.002)	-0.031 (0.002)
Age	0.000 (0.001)	0.000 (0.001)	-0.000 (0.000)	-0.000 (0.000)	0.000 (0.000)	-0.000 (0.000)	0.005 (0.001)	0.005 (0.001)
AgeS	0.000 (0.001)	0.000 (0.001)	0.000 (0.000)	0.000 (0.000)	0.000 (0.000)	0.000 (0.000)	-0.000 (0.000)	-0.000 (0.000)
Married	0.002 (0.001)	0.002 (0.001)	0.000 (0.001)	0.000 (0.001)	0.002 (0.001)	0.000 (0.001)	0.014 (0.002)	0.014 (0.002)
Childreen	-0.001 (0.001)	-0.001 (0.001)	-0.001 (0.001)	-0.001 (0.001)	-0.001 (0.001)	-0.001 (0.001)	0.003 (0.002)	0.003 (0.002)
Uni_Educ	0.001 (0.001)	0.001 (0.001)	-0.003 (0.001)	-0.003 (0.001)	0.001 (0.001)	-0.003 (0.001)	0.018 (0.002)	0.018 (0.002)
Employment	-0.008 (0.002)	-0.008 (0.002)	-0.001 (0.001)	-0.001 (0.001)	-0.008 (0.002)	-0.001 (0.001)	-0.013 (0.004)	-0.013 (0.004)
lH_Income	-0.005 (0.001)	-0.005 (0.001)	-0.002 (0.000)	-0.002 (0.000)	-0.005 (0.001)	-0.002 (0.001)	0.002 (0.003)	0.001 (0.003)
lH_Asset	0.001 (0.001)	0.001 (0.001)	-0.001 (0.001)	-0.002 (0.001)	0.001 (0.001)	-0.002 (0.000)	0.010 (0.002)	0.010 (0.002)
Risk_Aversion	0.012 (0.002)	0.012 (0.002)	0.006 (0.001)	0.006 (0.001)	0.012 (0.002)	0.006 (0.000)	0.006 (0.004)	0.006 (0.004)
Myopic_View	0.001 (0.001)	0.002 (0.001)	0.001 (0.000)	0.001 (0.000)	0.001 (0.000)	0.001 (0.000)	-0.000 (0.001)	-0.000 (0.001)
Urban_Dummey	0.002 (0.001)	0.002 (0.001)	0.002 (0.001)	0.001 (0.001)	0.002 (0.001)	0.002 (0.001)	-0.004 (0.003)	-0.004 (0.003)

## Supporting information

S1 Data Original data used in the analysis
(XLSX)
